# The application of network label propagation to rank biomarkers in genome-wide Alzheimer’s data

**DOI:** 10.1186/1471-2164-15-282

**Published:** 2014-04-14

**Authors:** Matthew E Stokes, M Michael Barmada, M Ilyas Kamboh, Shyam Visweswaran

**Affiliations:** 1 Department of Biomedical Informatics, University of Pittsburgh, 5607 Baum Boulevard, 15206 Pittsburgh, PA, USA; 2 The Intelligent Systems Program, University of Pittsburgh, Pittsburgh, PA, USA; 3 Department of Human Genetics, University of Pittsburgh, Pittsburgh, PA, USA

**Keywords:** Bioinformatics, Genome-wide association study, Feature ranking, Label propagation, Prediction, Reproducibility, Single nucleotide polymorphism, Alzheimer’s disease

## Abstract

**Background:**

Ranking and identifying biomarkers that are associated with disease from genome-wide measurements holds significant promise for understanding the genetic basis of common diseases. The large number of single nucleotide polymorphisms (SNPs) in genome-wide studies (GWAS), however, makes this task computationally challenging when the ranking is to be done in a multivariate fashion. This paper evaluates the performance of a multivariate graph-based method called label propagation (LP) that efficiently ranks SNPs in genome-wide data.

**Results:**

The performance of LP was evaluated on a synthetic dataset and two late onset Alzheimer’s disease (LOAD) genome-wide datasets, and the performance was compared to that of three control methods. The control methods included chi squared, which is a commonly used univariate method, as well as a Relief method called SWRF and a sparse logistic regression (SLR) method, which are both multivariate ranking methods. Performance was measured by evaluating the top-ranked SNPs in terms of classification performance, reproducibility between the two datasets, and prior evidence of being associated with LOAD.

On the synthetic data LP performed comparably to the control methods. On GWAS data, LP performed significantly better than chi squared and SWRF in classification performance in the range from 10 to 1000 top-ranked SNPs for both datasets, and not significantly different from SLR. LP also had greater ranking reproducibility than chi squared, SWRF, and SLR. Among the 25 top-ranked SNPs that were identified by LP, there were 14 SNPs in one dataset that had evidence in the literature of being associated with LOAD, and 10 SNPs in the other, which was higher than for the other methods.

**Conclusion:**

LP performed considerably better in ranking SNPs in two high-dimensional genome-wide datasets when compared to three control methods. It had better performance in the evaluation measures we used, and is computationally efficient to be applied practically to data from genome-wide studies. These results provide support for including LP in the methods that are used to rank SNPs in genome-wide datasets.

## Background

The volume of genomic data generated from genome-wide association studies (GWASs) is growing at an exponential rate, in large part due to the decreasing cost of high-throughput genotyping technologies. A GWAS measures hundreds of thousands of single nucleotide polymorphism (SNPs) across the human genome; a SNP is the commonest type of genetic variation that results when a single nucleotide is replaced by another in the genome sequence. The goal of a GWAS is typically biomarker discovery, that is, to discover SNPs that either singly or in combination are associated with the disease of interest. The high dimensionality of GWAS data poses statistical and computational challenges in identifying associations between SNPs and disease efficiently and accurately.

The typical analysis of GWAS data involves the application of a univariate feature ranking method that evaluates each SNP’s strength of association with disease independently of all other SNPs. For example, the chi squared statistic is used to assess the expected and observed genotypes of a SNP in cases and controls in a GWAS, and ranks SNPs according to the p-value. Univariate methods have the advantage of being are computationally efficient; however, they cannot capture interactions among genes and such interactions may play an important role in genetic basis of disease. Moreover, univariate methods may be associated with lack of reproducibility across datasets; that is, SNPs found to be relevant in one study do not show an association in another. Multivariate feature ranking methods evaluate each SNP’s strength of association with disease in the context of other SNPs. For example, Relief is capable of detecting complex SNP-SNP dependencies even in the absence of main effects. However, multivariate methods are computationally demanding since they consider the strength of association with disease of combinations of SNPs.

This paper describes the application of an efficient and stable multivariate machine learning method called label propagation (LP). LP has been applied successfully on other types of biological data, including gene expression and protein concentration data [1]; however, to our knowledge, the method has not been applied to datasets with a very large number of features as found in GWASs. We apply LP to two Alzheimer’s disease GWAS datasets. We conjectured that it would be efficient, produce reproducible rankings of SNPs and perform well. A positive result would support using LP in analyzing other genome-wide datasets, including next-generation genome-wide datasets that contain even larger number of SNPs.

The following sections provide background information about genome-wide association studies, feature ranking methods, and Alzheimer’s disease.

### Genome-wide association studies

In a GWAS, high-throughput genotyping technologies are used to assay hundreds of thousands or even millions of SNPs across the genome in a cohort of cases and controls. Since the advent of GWASs many common diseases, including Alzheimer’s disease, diabetes, and heart disease have been studied with the goal of identifying the underlying genetic variations. The success of GWASs in identifying genetic variants associated with a disease rests on the common disease-common variant hypothesis. This hypothesis posits that common diseases are caused usually by relatively common genetic variants and individually many of these variants have low penetrance and hence have small to moderate association with the disease [2].

In the past decade, GWASs have been moderately successful and have identified approximately 4,500 common disease-associated SNVs, and several hundred of the SNVs have been replicated [3]. A possible reason for the moderate success of GWASs is the common disease-rare variant hypothesis, which posits that many rare variants underlie common diseases and each variant causes disease in relatively few individuals with high penetrance [2]. However, larger sample sizes and new analytical methods will likely make GWASs useful for detecting rare variants as well [4].

### Feature selection and feature ranking methods

High-throughput genotyping and other biological technologies offer the promise of identifying sets of features that represent biomarkers for use in biomedical applications. The challenge with these high-dimensional data is that the selection of a small set of features or the ranking of all features requires robust feature selection and feature ranking methods.

A range of selection and feature ranking methods have been developed and a recent review of the methods is provided in [5]. There are two major families of feature selection methods, namely, filter methods and wrapper methods. Filter methods evaluate features directly independent of how the features will be used subsequently. In contrast, wrapper methods evaluate features in the context of the how they will be used. For example, if features are to be used subsequently to develop a classification model, a wrapper method evaluates the goodness of features in terms of their ability to improve the performance of the classification model.

Filter methods assess the relevance of features by examining only the intrinsic properties of the data. Univariate filter methods compute the relevance of each feature independently of other features. They are computationally fast and scale to high-dimensional data because the complexity is linear in the number of features and interactions between features are ignored. Typically, such methods compute a statistic or a score for each feature such as chi squared or information gain. Multivariate filter methods model correlations and dependencies among the features; they are computationally somewhat slower and may be less scalable to high-dimensional data. Examples of multivariate methods include correlation-based feature selection and Markov blanket feature selection.

The chi squared statistic is commonly used in SNP analysis is a univariate filter method. This test measures whether outcome distributions are significantly different among SNP states, indicating features that have an impact on disease. The chi squared statistic is very fast to compute and has a simple statistical interpretation. However, it cannot detect higher-order effects such as SNPs that interact to produce an effect on disease.

The Relief method [6] is a multivariate filter method that has been applied to SNP data to rank SNPs. This method computes the relevance of a SNP by examining patterns in a local neighborhood of training samples. The method examines whether, among reasonably similar samples, a change in SNP state is accompanied by a change in the disease state. Relief can detect multivariate interaction effects by means of the neighborhood locality measure, but does so at the cost of increased computation time. Relief has been adapted in several ways for application to SNP data. The most recently described adaptations of Relief include Spatially Uniform ReliefF (SURF) [7,8] and Sigmoid Weighted ReliefF (SWRF) [9] that were developed specifically for application to high-dimensional SNP data.

Logistic regression is another commonly used multivariate method that has been applied to many bioinformatics tasks for both classification and feature ranking. More recently, sparse logistic regression (SLR) models which are implicitly feature-selective have been developed for high-dimensional data. SLR uses L1-norm regularization that drives the weights of many of the features to zero, and has been used successfully as a feature selection method in high-dimensional biomedical data, including fMRI imaging data [10] and genomic data [11].

One challenge of feature ranking in genomic data arises from the observation that a group of SNPs that are in linkage disequilibrium (LD) will be statistically correlated and can lead to redundancy when many of the top-ranked variants represent the same genetic signal. This is particularly an issue with univariate tests like chi squared, which operate solely on observational data counts. With such a test, SNPs that have near-identical case–control distributions will be assigned near-identical scores. The problem is mitigated somewhat by multivariate methods that utilize locality or other inference. By considering the context of each attribute, even SNPs with near-identical case–control distributions may be assigned different scores based on the context of surrounding SNPs.

### Alzheimer’s disease

Alzheimer’s disease (AD) is a neurodegenerative disease characterized by slowly progressing memory failure, confusion, poor judgment, and ultimately, death [12]. It is the most common form of dementia associated with aging. There are two forms of AD, called familial AD and sporadic AD. The rarer form is early-onset familial AD, which typically begins before 65 years of age. The genetic basis of early-onset AD is well established, and it exhibits an autosomal dominant mode of inheritance. Most familial cases of AD are accounted for by mutations in one of three genes (amyloid precursor protein gene, presenelin 1 or presenelin 2).

Sporadic AD, also called late-onset AD (LOAD), is the commoner form of AD, accounting for approximately 95% of all AD cases. The onset of LOAD symptoms typically occurs after 65 years age. LOAD has a heritable component, but has a more genetically complex mechanism than familial AD. The strongest consistently replicated genetic risk factor for LOAD is the apolipoprotein E (APOE) gene. Two genetic loci (rs429358 and rs7412) together determine the allele of the APOE gene, which are called APOE*2, APOE*3 and APOE*4. The APOE*4 allele is a LOAD risk factor, while the APOE*2 allele is associated with reduced risk [13].

In the past several years, GWASs have identified several additional genetic loci associated with LOAD. Over a dozen significantly associated loci have been published in the literature, resulting from meta-analyses of several AD GWASs [14-16].

### Label propagation

This section first provides an overview of the label propagation and then provides more details of the method.

### Overview

LP is a machine learning method that can be used for prediction (e.g., predicting case/control status from SNP measurements on a sample) and as a multivariate feature ranking method (e.g., ranking SNPs in a case/control GWAS dataset). It is graph-based algorithm that represents the data as a bipartite graph. A bipartite graph contains two sets of nodes (i.e., sample nodes that represent individuals and feature nodes that represent SNPs in GWAS data) and edges that link nodes from one set to nodes in the other set. The sample nodes are labeled with case/control status, and LP diffuses the labels across graph edges to the feature nodes and back again, until a stable solution is reached. The solution results in a final labeling of all nodes in the graph, including the feature nodes, which balances the diffusion of the labels with consistency with the original labeling. The labeling of the feature nodes can be used to rank the features, and the labeling of the sample nodes can be used as predictions.

The LP method scales well for thousands of samples and features. It has complexity *O*(*kNF*), where *N* is the number of samples, *F* is the number of features and *k* is the number of iterations required for convergence. Typically, *k* is much smaller than *N* or *F*, which makes LP a relatively fast method. LP is able to handle missing data and both continuous and discrete data.

Because of its wide applicability, fast running time, and multivariate nature, LP has been applied to several bioinformatics problems. LP has been used in breast cancer gene expression data in order to find functional modules of co-expressed genes [17]. It has been applied to gene function prediction, utilizing known gene functions and interactions to infer the function of other genes [18]. It has shown success in classifying patients with Alzheimer’s disease using protein array data [19]. To our knowledge, LP has not been applied to SNP data. Unique challenges in the SNP domain include a much larger feature space (on the order of hundreds of thousands), as well as the discrete, nominal nature of SNP states (as opposed to the continuous nature of expression data).

### Algorithmic details

**Figure 1:**
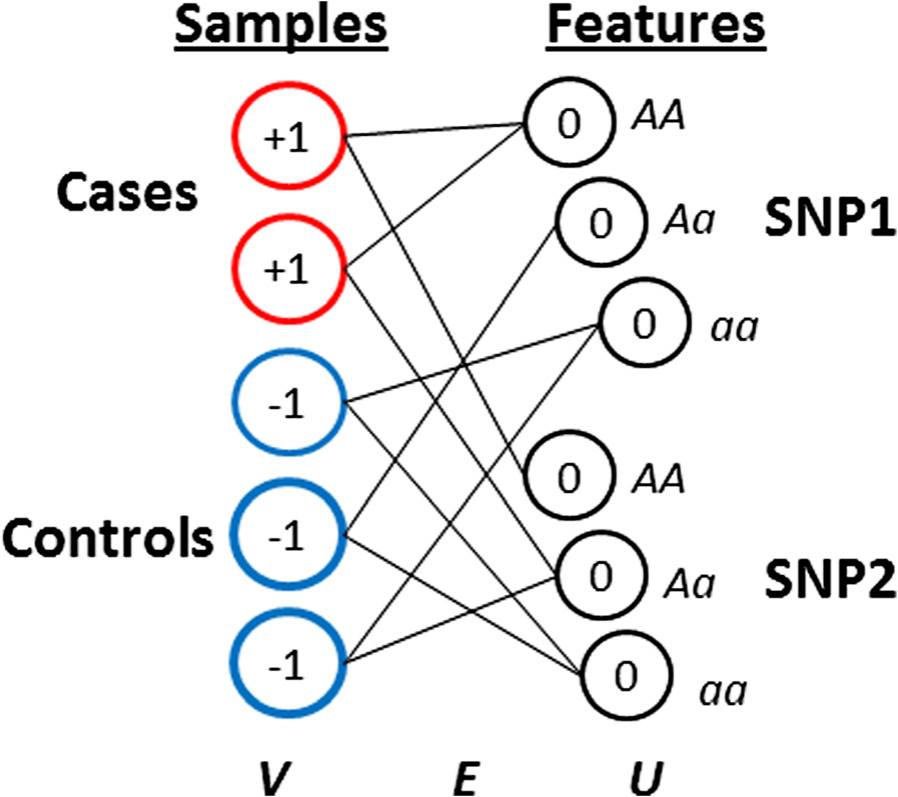
**A small bipartite graph for a hypothetical dataset with five samples and two SNPs.** The five samples are represented by the nodes at the left (*V*), and are labeled with case or control status (+1, -1, respectively). Each SNP is represented by three nodes at the right (*U*) with one node for each SNP state (AA = major homozygote, Aa = heterozygote, aa = minor homozygote). Edges represent actual observations in the dataset and connect samples to the SNP states that they exhibit. Labels are allowed to propagate along edges and result in a final labeling for each node in the range (-1, +1), indicating association with case or control status.

We represent a GWAS dataset as a bipartite graph *G =* (*V, U, E*) which consists of two sets of nodes *V* and *U* where nodes in *V* represent samples (individuals) and nodes in *U* represent features (SNP states). Note that if a SNP has three states (major homozygote, heterozygote and minor homozygote) than it will be represented by three nodes in *U*. In addition to the two sets of nodes, the graph contains a set of edges *E* where each edge links a node in *V* with a node in *U*. An edge *E*(*v,u*) that links node *v* with node *u* is associated with a link weight *w*(*v, u*) =1. These edges connect sample nodes to feature nodes, representing the presence of SNP state *u* in individual *v*. Initial labels *y(v)* and *y(u)* are applied to nodes, and take values {-1, 0, +1}, representing known training information about case/control status (+1 and -1, respectively), or a lack of information (0). An example graph initialization is shown in Figure 1.

Given the graph initialization, the propagation algorithm finds an optimal assignment of node labels *f*(*v*) and *f*(*u*), which minimizes the objective function

$$\begin{array}{ll} Q\left(f\right)= & \sum_{\left(v,u\right)\in E}w\left(v,u\right){\left(\frac{f\left(v\right)}{\sqrt{d\left(v\right)}}-\frac{f\left(u\right)}{\sqrt{d\left(u\right)}}\right)}^{2}\\ & +\mu \left(\sum_{v\in V}{\left(f\left(v\right)-y\left(v\right)\right)}^{2}+\sum_{u\in U}{\left(f\left(u\right)-y\left(u\right)\right)}^{2}\right) \end{array}$$

where *μ* is a parameter controlling the relative effect of the two parts of the cost function.

The first part of the equation is a smoothness constraint, ensuring that strongly connected nodes in *V* and *U* get similar labels. Here, *d*(*v*) and *d*(*u*) are the degree of each node in *V* and *U*, such that *d*(*v*) = ∑ _(*v*,*u*) ∈ *E*_*w*(*v*, *u*) and *d*(*u*) = ∑ _(*v*,*u*) ∈ *E*_*w*(*v*, *u*). The second part of the equation is a fitting constraint. For labeled nodes, this ensures that nodes labels are consistent with the initial labeling. For unlabeled nodes, this term constrains the overall cost. In the discrete-label case where *f* → {-1, 0, +1}, the optimization of this cost function is NP-hard. By relaxing the labels so that *f* → *R*, however, the optimization of this equation becomes straightforward as derived in Zhou [20], and has the solution *f*^∗^ = (1 - *α*)(*I* - *αS*)^- 1^*Y*. Here, *I* is the identity matrix and *S* is the normalized connectivity matrix $S=\left[\begin{array}{cc} 0 & D_{V}^{-1/2}WD_{U}^{-1/2}\\ D_{U}^{-1/2}W^{T}D_{V}^{-1/2} & 0 \end{array}\right]$, where *W* is the |*V*| × |*U*| sized matrix of edge weights and *D*_*V*_ and *D*_*U*_ are the |*V*| × |*V*| and |*U*| × |*U*| diagonal matrices containing node degrees, respectively.

While the solution may be computed directly by algebraic evaluation, it requires the inversion of a *T* × *T* matrix where *T* is the total number of nodes in the network (*T* = |*V*| + |*U*|). This requires between *O*(*T*^*2*^) and *O*(*T*^*3*^) time, depending on the inversion method used. Instead, we use an iterative procedure that diffuses node labels from one node set to another. First, the normalized graph Laplacian is computed as B=DV-12WDU-12. This is a special encoding of the graph which represents node degrees and adjacency. It has an interpretation as a random walk transition matrix, allowing labels to travel across graph edges. The node labels on *V* and *U* are computed iteratively as

$$\begin{array}{l} f_{t+1}\left(V\right)=\left(1-\alpha \right)y\left(V\right)+\alpha Bf_{t}\left(U\right)\;\text{and}\\ f_{t+1}\left(U\right)=\left(1-\alpha \right)y\left(U\right)+\alpha Bf_{t}\left(V\right) \end{array}$$

where *α* is a user-specified parameter in the range [0, 1] that controls the balance between the initial labeling *y* and the diffusion of current labels *f*. This procedure ultimately converges to the same optimized node labeling as the direct algebraic evaluation. The complexity of the direct algebraic evaluation is at least *O*((|*V*| + |*U*|)^2^), while the complexity of the iterative procedure is *O*(*k*|*V*||*U*|), where *k* is the number of iterations required for convergence. The exact value of *k* depends on the properties of the graph as well as the convergence criteria, but was found to be orders of magnitude less than both *|V|* and *|U|* even when analyzing large graphs (>100,000 nodes) with large alpha (>0.9).

The final labeling of the nodes indicates association with the case or control group. Nodes with scores near +1 are associated with the case group, nodes with scores near -1 are associated with the control group, and nodes with scores near 0 are uninformative. For sample nodes, this score can be viewed as a prediction of case/control status based on genetic information. For feature nodes, this score can be interpreted as an association test that can be used to find biologically significant markers. The feature node scores may be ordered to obtain a ranking of feature according to their association with the outcome.

## Methods

This section provides details of the datasets and the experimental design, the evaluation measures we used to evaluate LP, and the comparison methods including chi squared, SWRF, and SLR.

### Datasets

#### ***Synthetic dataset***

We created a synthetic dataset containing 1,000 SNPs and a binary phenotype that is a function of 35 of those SNPs (“causal” SNVs). Of the 35 SNPs, 10 of them were modeled as more common SNPs with MAFs that were sampled uniformly from the range 0.0500 to 0.5000 with odds ratios in the range 1.05 to 1.50 and 25 SNPS were modeled as rare SNPs that were sampled uniformly from the range 0.0001 to 0.0100 and odds ratios in the range 2 to 10. The remaining 965 SNVs (“noise” SNVs) ranged from common to rare, but do not have an effect on the disease. Phenotype status was assigned using an additive threshold model, with each causal SNP conferring an independent risk of disease. We created a set of 1,000 individuals and in that set 13.3% of individuals had a positive phenotype. The comparable number of samples and features make this model fairly robust to variations across instantiations of the data, reducing the need for multiple runs to observe “average” statistical performance.

#### ***GWAS datasets***

We used two different LOAD GWAS datasets. The first dataset comes from the University of Pittsburgh Alzheimer’s Disease Research Center (ADRC) [21]. This dataset consists of 2,229 individuals of which 1,291 were diagnosed with LOAD and 938 were healthy age-matched controls. In the original study 1,016,423 SNPS were measured and after quality controls were applied by the original investigators 682,685 SNPs located on autosomal chromosomes were retained for analysis.

The second dataset comes from the Translational Genomics Research Institute (TGen) located in Phoenix, Arizona [22]. This dataset consists of three cohorts containing a total of 1,411 individuals of which 861 were diagnosed with LOAD and 550 were healthy age-matched controls. In the original study 502,627 SNPs were measured for each individual and after quality controls were applied by the original investigators 234,665 autosomal SNPs were retained for analysis.

Principal components analysis of each dataset indicated no significant population stratification between the cases and the controls. Between the datasets, however, differing allele frequencies are exhibited as indicated by clustering in the principal components analysis of the combined data. Because of this, we do not combine the datasets for a unified analysis, but still use cross-dataset learning to test generalizability of results.

For the ranking reproducibility and cross-dataset classification experiments, we retained from both datasets only those SNPs that were measured in both studies. There are 64,984 SNPs that are common across the two datasets. In addition, we performed smaller-scale experiments on SNPs from chromosome 19, which is known to contain several genetic variants that are associated with LOAD. There are 13,087 SNPs in chromosome 19 in the ADRC dataset and 3,652 SNPs in the TGen dataset, and 1,307 SNPs are common across the two datasets.

### Experimental methods

We compared the performance of LP to the performance of three control methods, which were chi squared, SWRF, and SLR. We applied the four methods to the synthetic data to rank SNPs associated with the phenotype. After ranking, we plotted precision-recall and ROC curves to examine how well the truly associated SNPs were ranked.

For the real data, we applied each method to two GWAS datasets to rank SNPs that are predictive of LOAD. We performed the experiments on a small-scale subset of the data consisting of only those SNPs in chromosome 19 which contains several well established LOAD-related SNPs, and on the full genome-wide data. We evaluated the rankings produced by the four methods by classification performance and feature reproducibility across the two datasets. In addition, we examined the top-ranked SNPs from each method for previous evidence in the literature that they are associated with LOAD.

### Classification performance

Meaningful features should be predictive of disease, and classifiers developed from highly predictive SNPs should have good performance in discriminating between cases and controls. We evaluated the predictive performance of the top-ranked SNPs for each feature ranking method and dataset by measuring the performance of a series of classification models that were developed using progressively larger number of top-ranked SNPs. Given a set of top-ranked SNPs obtained from a ranking method applied to a training dataset, we applied the *k*-nearest neighbor (*k*NN) classification method to a test dataset containing genotypes for the corresponding SNPs. We evaluated the performance of *k*NN using fivefold cross-validation. The dataset was randomly partitioned into five approximately equal sets such that each set had a similar proportion of individuals who developed LOAD. We applied the ranking method on four sets taken together as the training data, and evaluated the classifer performance of the top-ranked SNPs on the remaining test data. We repeated this process for each possible test set to obtain a LOAD prediction for each individual in the dataset. We used the predictions to compute the area under the Receiver Operating Characteristic curve (AUC) which is a widely used measure of classification performance.

In addition, we performed cross-dataset validation experiments on the filtered dataset containing the common SNPs. Here, SNPs were ranked on one dataset, and the top-ranked SNPs were used to derive a *k*NN classifier on the other dataset. These experiments show the generalizability and robustness of the methods, quantifying how well inference on one dataset can be applied to another cohort.

The LP method is presented with a parameterization of α = 0.25. This parameterization was selected after testing several values between 0.1 and 0.9 on the small-scale TGen dataset. The setting of 0.25 puts more emphasis on matching the case/control training labels while still utilizing some network diffusion, and is suitable for finding discriminative SNPs. Smaller values of alpha lead to rankings that are indistinct from the chi squared test, while larger values lead to uninformative, uniform feature scores.

### Feature reproducibility

With the predictive power of the top-ranked SNPs established, we evaluated the feature ranking methods for reproducibility across the two datasets. For the genome-wide datasets, we reduced them so that they contained only the genotypes for the 64,984 SNPs that were common to both. We ran each feature ranking method separately on each of the reduced datasets and examined the ranked SNPs for reproducibility. Given two ranked list of SNPs obtained by applying a feature ranking method to the two reduced datasets we examined the ranked lists for common SNPs in the top-ranked 10 SNPs, 50 SNPs, 100 SNPs, and so on. Reproducibility was calculated as the number of SNPs in common to both lists divided by the total number of SNPs in a list, yielding a value in the range from 0 (no SNPs in common) to 1 (both lists contain exactly the same SNPs). This metric only checks for presence or absence of SNP in a list, and ignores actual ranks within the list. The LP method is presented for multiple setting of α, ranging from 0.25 to 0.9.

### Evidence from the literature

We examined the top-ranked SNPs for biological significance and evidence of previously documented association with LOAD. We used several publically available databases and resources including SNPedia [23], GeneCards [24], and dbSNP [25] to search for links between the variants and LOAD. In addition to SNPs directly named in the literature as having an association with LOAD, we also considered a wider degree of plausible associations. For each SNP, we searched whether it was in strong linkage disequilibrium with LOAD-related SNPs, whether the SNP was in a LOAD-related gene, whether the associated gene was part of a strongly conserved, LOAD-related family, or whether the variant has been associated with brain development or other neurological conditions.

### Computational efficiency

We ran all four methods on a PC with a 2.33 GHz Intel processor and 4 GB of RAM. All methods were implemented in Java, except for the SLR method, which is a MATLAB package [10]. For each feature ranking method, we recorded the time required to score features on one training fold of the ADRC dataset.

## Results

### Synthetic data

**Figure 2:**
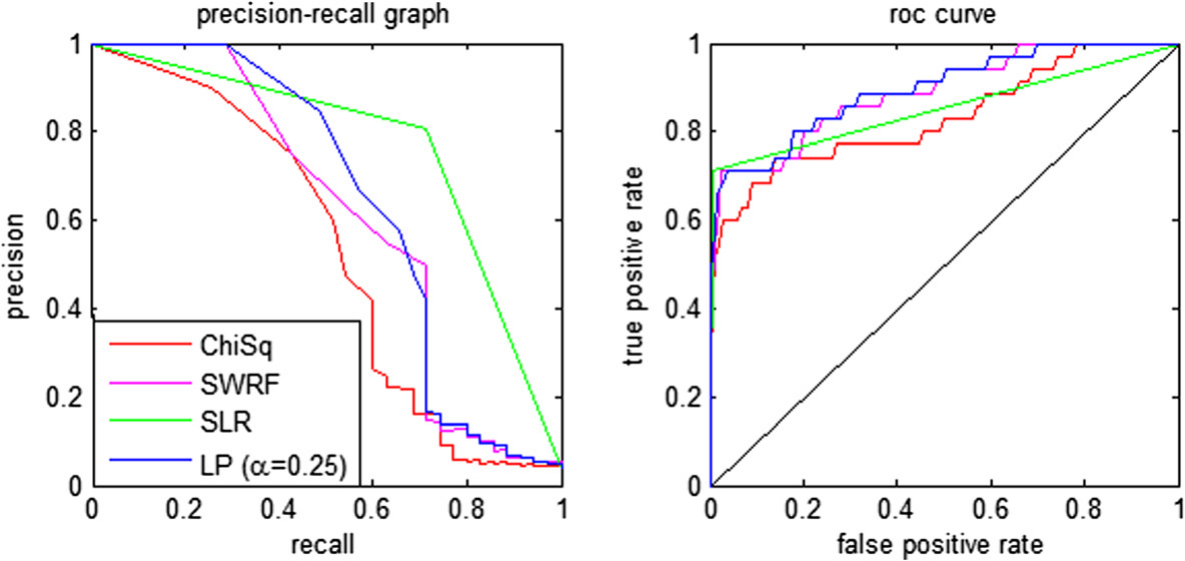
**Precision-recall and ROC curves for four feature ranking methods on synthetic data.** There are 35 true phenotype-associated SNPs in this dataset.

Figure 2 shows the precision-recall and ROC curves obtained from the four ranking methods on the synthetic dataset. All methods do quite well in retrieving the 35 causal SNPs. The SLR method performs the best on this dataset, showing excellent retrieval even for small-effect SNPs. The other methods perform well, identifying nearly all of the large-effect SNPs at the top of the ranking. The small-effect size SNPs fall somewhat lower in the ranking, as indicated by the tail in the precision-recall graphs for three of the methods. All four methods perform similarly in the ROC space, achieving similar true positive rates for a given false positive rate. The shape of the ROC graph again indicates that all of the methods rank most of the valid SNPs at the top of the list, but only find the small-effect size SNPs after many false positives. These results provide support that each of the methods is able to find valid associations in SNP data over a range of MAFs and effect sizes.

### GWAS classification performance

**Figure 3:**
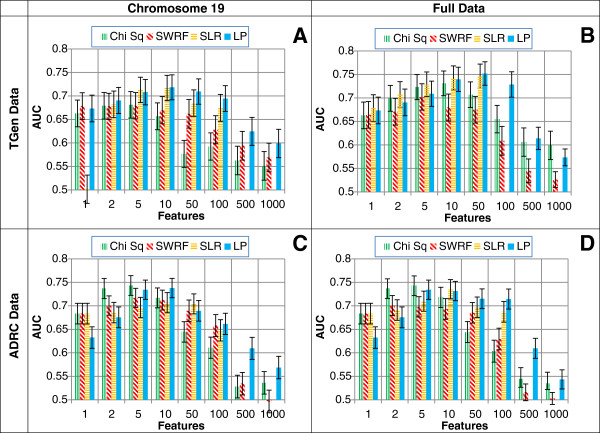
**AUCs with 95% confidence intervals for four different feature ranking methods (chi square, SWRF, SLR, and LP) obtained from application of the *k*NN classifier on two LOAD datasets (TGen and ADRC) with two sets of SNPs (chromosome 19 only and genome-wide).** The datasets used in the four panels are: **A)** TGen chromosome 19 only (3,652 SNPs); **B)** TGen genome-wide (234,665 SNPs); **C)** ADRC chromosome 19 only (13,087 SNPs); **D)** ADRC genome-wide (682,685 SNPs). The SLR method implicitly selects <500 features in each experiment.

Figure 3 shows the AUCs obtained for the four ranking methods obtained from application of the *k*NN classifier on the two LOAD datasets. Generally, LP achieves equal or higher AUCs than chi squared and SWRF, and similar AUCs to SLR. On the small-scale datasets (containing chromosome 19 SNPs only) LP achieves statistically significantly higher AUCs at the 5% significance level when compared to chi squared and SWRF in the range from 10 to 1000 top-ranked SNPs (see Table 1). On the genome-wide datasets, similar statistically significantly higher AUCs were achieved by LP in the range from 50 to 100 top-ranked SNPs (see Table 1). LP has a statistically significantly lower AUC than chi squared or SWRF in only two experiments, when using just 1 or 2 SNPs in the ADRC dataset. When using at least 10 SNPs, LP always significantly outperforms either chi squared or SWRF, or both. The SLR method does not perform significantly differently from LP in all experiments that use more than one SNP for classification.

**Table 1:** Prediction results for feature ranking methods (chi squared, SWRF, SLR and LP) on two LOAD datasets (TGen and ADRC) with two sets of SNPs (chromosome 19 only and genome-wide)

**Dataset**	**# SNPs**	**Method**	**Number of SNPs used in classifier**							
			**1**	**2**	**5**	**10**	**50**	**100**	**500**	**1000**
TGen	3,652 (chr19)	Chi Sq	0.6628 ± 0.0284	0.6791 ± 0.0278	0.6811 ± 0.0280	0.6567 ± 0.0290	0.5768 ± 0.0306	0.5925 ± 0.0300	0.5627 ± 0.0306	0.5512 ± 0.0304
		SWRF	0.6783 ± 0.0284	0.6778 ± 0.0284	0.6791 ± 0.0282	0.6697 ± 0.0286	0.6620 ± 0.0286	0.6280 ± 0.0290	0.5941 ± 0.0304	0.5691 ± 0.0306
		SLR	0.5014 ± 0.0302	0.6821 ± 0.0282	0.7129 ± 0.0270	0.7170 ± 0.0269	0.6847 ± 0.0282	0.6747 ± 0.0286	*	*
		LP	0.6733 ± 0.0284	0.6904 ± 0.0278	0.7080 ± 0.0270	**0.7184 ± 0.0267**	**0.7093 ± 0.0270**	**0.6945 ± 0.0274**	**0.6246 ± 0.0298**	**0.5989 ± 0.0302**
	234,665 (chr1-22)	Chi Sq	0.6628 ± 0.0284	0.6991 ± 0.0274	0.7230 ± 0.0267	0.7310 ± 0.0263	0.7068 ± 0.0272	0.6549 ± 0.0292	0.6059 ± 0.0302	0.5990 ± 0.0302
		SWRF	0.6640 ± 0.0284	0.6705 ± 0.0284	0.7020 ± 0.0280	0.6796 ± 0.0286	0.6749 ± 0.0284	0.6087 ± 0.0306	0.5447 ± 0.0253	0.5261 ± 0.0169
		SLR	0.6783 ± 0.0284	0.7076 ± 0.0270	0.7291 ± 0.0261	0.7424 ± 0.0257	0.7464 ± 0.0257	*	*	*
		LP	0.6733 ± 0.0284	0.6904 ± 0.0284	0.7088 ± 0.0269	**0.7396 ± 0.0257**	**0.7519 ± 0.0251**	**0.7286 ± 0.0270**	**0.6138 ± 0.0237**	**0.5735 ± 0.0178**
ADRC	13,087 (chr19)	Chi Sq	0.6834 ± 0.0220	0.7369 ± 0.0206	0.7433 ± 0.0204	0.7169 ± 0.0212	0.6446 ± 0.0229	0.6109 ± 0.0233	0.5282 ± 0.0239	0.5361 ± 0.0241
		SWRF	0.6834 ± 0.0229	0.7006 ± 0.0221	0.7169 ± 0.0206	0.7122 ± 0.0206	0.6894 ± 0.0220	0.6580 ± 0.0225	0.5343 ± 0.0235	0.4965 ± 0.0239
		SLR	0.6834 ± 0.0220	0.6855 ± 0.0218	0.6964 ± 0.0216	0.7068 ± 0.0213	0.7041 ± 0.0213	0.6478 ± 0.0227	*	*
		LP	0.6325 ± 0.0220	0.6756 ± 0.0214	0.7342 ± 0.0210	0.7378 ± 0.0212	**0.6894 ± 0.0218**	**0.6616 ± 0.0225**	**0.6095 ± 0.0241**	**0.5687 ± 0.0239**
	682,685 (chr1-22)	Chi Sq	0.6834 ± 0.0220	0.7369 ± 0.0206	0.7433 ± 0.0204	0.7184 ± 0.0212	0.6438 ± 0.0227	0.6034 ± 0.0235	0.5445 ± 0.0239	0.5349 ± 0.0239
		SWRF	0.6834 ± 0.0220	0.7006 ± 0.0213	0.6978 ± 0.0216	0.6934 ± 0.0220	0.6851 ± 0.0220	0.6293 ± 0.0231	0.5160 ± 0.0178	0.5029 ± 0.0127
		SLR	0.6834 ± 0.0220	0.6911 ± 0.0218	0.7100 ± 0.0214	0.7354 ± 0.0206	0.6970 ± 0.0218	0.6874 ± 0.0220	*	*
		LP	0.6325 ± 0.0229	0.6756 ± 0.0221	0.7342 ± 0.0206	0.7315 ± 0.0206	**0.7151 ± 0.0210**	**0.7145 ± 0.0214**	**0.6096 ± 0.0204**	**0.5435 ± 0.0122**

The entries are the cross-fold classification AUCs and the 95% confidence intervals obtained from application of the *k*NN classifier to a specified number of top-ranked SNPs. Bold cells indicate where LP significantly outperforms at least one of the other methods. The SLR method is implicitly feature-selective, reducing the feature space to under 500 features for all experiments (indicated by cells containing *).

For all four ranking methods, the classification performance shows the general trend of higher AUCs with a moderate number of SNPs used in the classifier, and lower AUCs at very small and very large numbers of SNPs. Compared to the other methods, LP’s performance drops far more slowly with increasing the number of SNPs in the classifier. This is a useful property for a feature ranking method, because the number of features to be used in a classifier can be a difficult number to choose. With too few features relevant SNPs may be missed, and with too many features irrelevant SNPs may be included. The LP method picks features which limit the amount of noise introduced, widening the useful performance range of the classifier. This reduces the chance of missing a relevant biomarker because of an overly restrictive feature selection threshold.

Results for the cross-dataset experiments are found in Table 2. Similar classification AUCs to the cross-validated experiments indicate that the selected features are robust between datasets, having meaning even in other patient cohorts. Several algorithms have trouble identifying a useful SNP in the #1 rank, possibly explained by stratification between the patient populations. Good performance is quickly achieved, however, providing further support that the selected variants are valid.

**Table 2:** Prediction AUCs for cross-dataset experiments

**Dataset**	**# SNPs**	**Method**	**Number of SNPs used in classifier**							
			**1**	**2**	**5**	**10**	**50**	**100**	**500**	**1000**
TGen (Feature selection from ADRC)	64,984 (ADRC overlap, chr1-22)	Chi Sq	0.6086 ± 0.0294	0.6863 ± 0.0280	0.7099 ± 0.0270	0.6958 ± 0.0253	0.6563 ± 0.0286	0.6097 ± 0.0296	0.5593 ± 0.0310	0.5563 ± 0.0308
		SWRF	0.5952 ± 0.0296	0.6980 ± 0.0274	0.6994 ± 0.0272	0.7005 ± 0.0274	0.6756 ± 0.0284	0.6677 ± 0.0284	0.5635 ± 0.0306	0.5195 ± 0.0310
		SLR	0.6086 ± 0.0294	0.6863 ± 0.0280	0.7164 ± 0.0269	0.7289 ± 0.0263	0.6522 ± 0.0292	0.6084 ± 0.0300	*	*
		LP	0.5023 ± 0.0306	0.6039 ± 0.0300	0.7023 ± 0.0272	0.7037 ± 0.0274	0.6888 ± 0.0276	0.6543 ± 0.0286	0.6114 ± 0.0298	0.5690 ± 0.0306
ADRC (Feature selection from TGen)	64,984 (TGen overlap, chr1-22)	Chi Sq	0.6172 ± 0.0231	0.6385 ± 0.0229	0.7419 ± 0.0204	0.7362 ± 0.0208	0.6695 ± 0.0225	0.6479 ± 0.0227	0.5396 ± 0.0239	0.5259 ± 0.0122
		SWRF	0.5397 ± 0.0239	0.5345 ± 0.0241	0.5350 ± 0.0241	0.5401 ± 0.0243	0.5042 ± 0.0243	0.5257 ± 0.0241	0.5201 ± 0.0241	0.5053 ± 0.0241
		SLR	0.5397 ± 0.0214	0.7006 ± 0.0214	0.7003 ± 0.0214	0.7048 ± 0.0216	0.6048 ± 0.0233	0.5854 ± 0.0237	*	*
		LP	0.5397 ± 0.0239	0.6021 ± 0.0235	0.7283 ± 0.0210	0.7366 ± 0.0208	0.6853 ± 0.0220	0.6598 ± 0.0225	0.5678 ± 0.0239	0.5306 ± 0.0239

The entries are the cross-dataset classification AUCs and the 95% confidence intervals. Feature selection was applied to one dataset, and the top-ranked features were used to derive and evaluate a *k*NN classifier on the other dataset. The SLR method is implicitly feature-selective, reducing the feature space to under 500 features for all experiments (indicated by cells containing *).

### GWAS feature reproducibility

**Figure 4:**
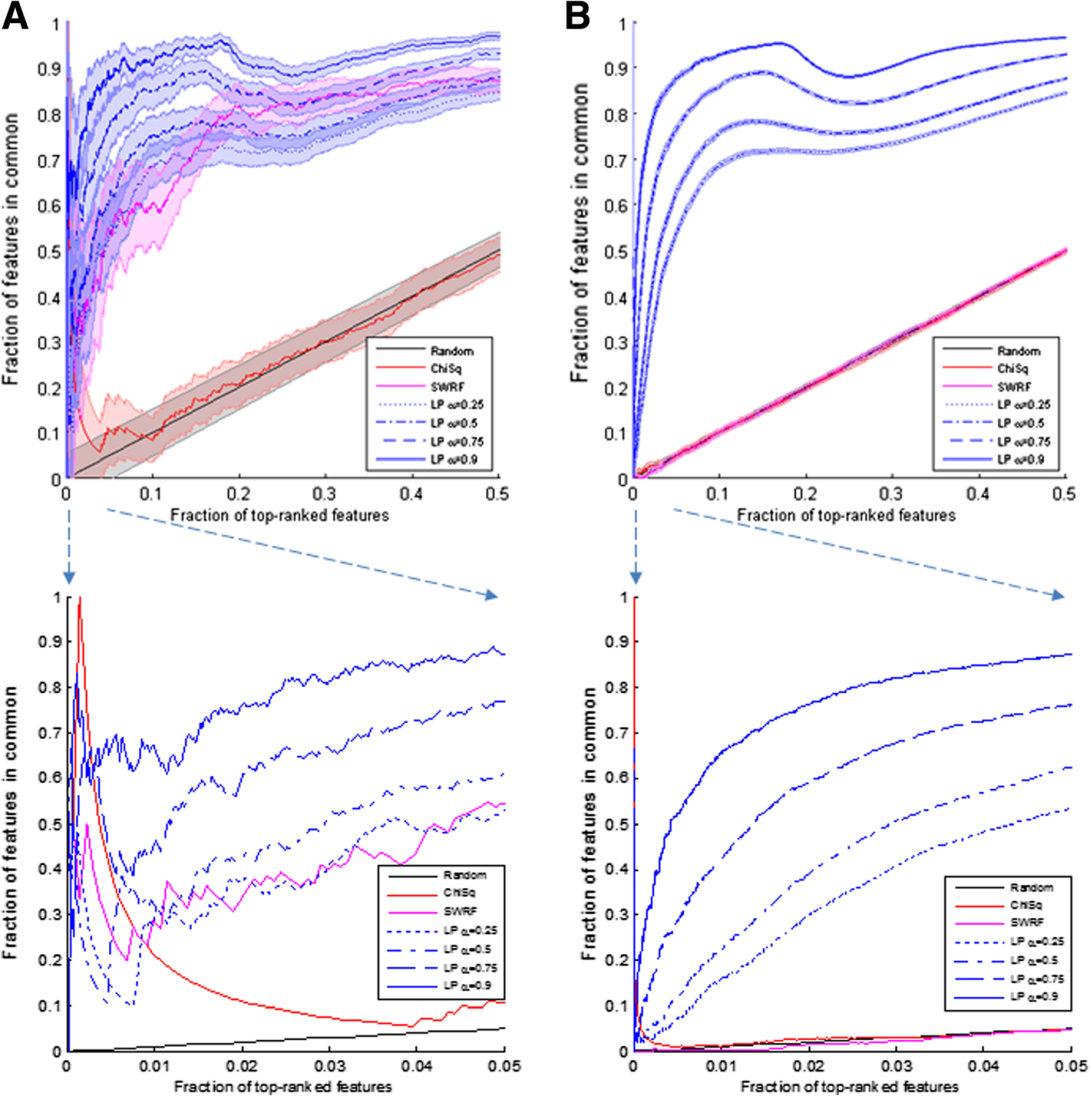
**Reproducibility curves of top-ranked features shown for top 50% of features (95% confidence interval), with callout for top 5% of features (CI omitted for clarity).** The x-axis shows the fraction of top-ranked features being considered, and the y-axis shows the fraction of features in common to rankings obtained from each of the two datasets independently (TGen and ADRC). The datasets used are: **A)** small-scale TGen and ADRC overlap data (chr19, 1,307 SNPs); **B)** genome-wide TGen and ADRC overlap data (chr 1–22, 64,984 SNPs). For this plot, the chi squared and SWRF methods are virtually indistinguishable from the random performance curve along the diagonal. SLR is omitted because it selects less than 0.5% of features with almost no reproducibility.

Figure 4 shows the reproducibility results on the small-scale and genome-wide datasets. Chi squared identifies the first few SNPs reproducibly; these are SNPs that are located in genes apolipoprotein-E (APOE) and apolipoprotein-C (APOC) and are known to have large effects sizes. Beyond the first few SNPs, however, the reproducibility of chi squared drops rapidly to a level which is effectively random. The SWRF method produces somewhat reproducible results in the small-scale chromosome 19 datasets, but is no better than random for the genome-wide datasets. The SLR method selects on the order of 100 SNPs for each filtered dataset, and is not shown on the reproducibility graph. For each SLR experiment, there are only two overlapping SNPs in each selected list, which are the two major loci on APOE. All other SNPs selected by SLR are not reproduced from one dataset to another. LP, in contrast to these methods, shows good reproducibility for many of the top-ranked SNPs, and does so even in the high-dimensional datasets. The method has low reproducibility for the first few SNPs but quickly surpasses chi squared, SWRF, and SLR. For higher values of α, LP has higher reproducibility. For α close to 0, diffusion of labels plays a small role in determining the ranking and LP behaves like a supervised method that computes a correlation measure. When α is close to 1, label diffusion has a greater effect on the ranking, and clusters in the data have a greater effect, yielding higher reproducibility. By utilizing the dense connectedness of nodes in modules of SNPs, LP produces more reproducible results.

### Evidence from the literature

Among the 25 top-ranked SNPs, several SNPs have previously known AD associations or have evidence for biologically plausibility of being involved in AD (see Additional file 1 for SNP lists). For both datasets, LP identified the highest number of plausibly associated SNPs. In the TGen dataset, 14 of top 25 SNPs identified by LP had evidence of being associated with LOAD, whether through direct association tests, co-location in associated genes, or through functional effects. In contrast, only 6 of the top 25 SNPs identified by chi squared had evidence of being associated with LOAD, SLR identified 7 associated SNPs, and SWRF identified 5 associated SNPs. In the ADRC dataset, 10 of the top 25 SNPs identified by LP had evidence of being associated with LOAD. Chi squared also identified 10 LOAD-related SNPs among the top 25; however, 7 of them are from a tightly clustered group of SNPs in chromosome 19 near the APOE locus, and do not represent a diverse genetic signal. SLR finds 5 associated SNPs in the ADRC data, and SWRF finds only 2. For both datasets, the remaining SNPs not found in the literature are generally located in relatively unstudied intergenic regions of the genome.

### Computational efficiency

Of the four ranking methods, chi squared is the fastest and took approximately 4 minutes to run on one training fold for the ADRC dataset. The SWRF method was the slowest and took almost 2 days to run. The SLR method was also slow, taking 11 hours and 29 minutes to complete. LP ran in 26 minutes for α = 0.25, and took 72 minutes for α = 0.9.

## Discussion

The results on the synthetic data show that LP performs comparably to the control methods that included chi squared, SWRF, and SLR. The SLR method performed particularly well in identifying small-effect SNPs in the synthetic data compared to LP and the other control methods. On the GWAS datasets, LP performed significantly better than chi squared and SWRF in terms of classification performance, reproducibility, and identified more SNPs among the top 25 ranked SNPs that had prior evidence of being associated with LOAD. When compared to SLR, LP had similar classification performance, but had better reproducibility and identified more SNPs among the top 25 ranked SNPs that had prior evidence of being associated with LOAD. In terms of computational efficiency LP is somewhat slower than chi squared, but is significantly faster than SWRF and SLR, and is sufficiently fast that it can be effectively applied to real genome-wide datasets. Overall, LP performs better than each of the control algorithms in one or more of the performance metrics tested, and does not perform significantly worse in any of them.

LP’s top-ranked features are reproducible across datasets, and provide good classification performance. The underlying genetic mechanisms and patterns of inheritance used in the graph-based LP method are also not as susceptible to changes in experimental protocol as more traditional methods. In contrast, chi squared computes a univariate statistic for each SNP, and is susceptible to errors in the data (misdiagnosed case/control status, misread genotype). LP, on the other hand, produces a score that depends on the distribution of all variants throughout the dataset. This score is not as susceptible to small errors because the largely correct training information is able to diffuse across the network and mitigate mistakes.

The network propagation method also allows for more diverse genetic signals to be scored highly. Chi squared ranks SNPs in strong LD closely together because it operates solely on the observational data counts. LP, on the other hand, can propagate influence from other parts of the network through sample nodes, meaning that even SNPs exhibited by mostly the same individuals can get different scores.

The LP method can be extended to handle significantly stratified data by using correction factors as described in [26]. In this method, principal components of variation are determined, and phenotypes and genotypes are adjusted to zero out this variation. Phenotype adjustment is simple, requiring only a re-labeling of the sample nodes. Genotype adjustment in LP is more complex, requiring edge weights other than 0 and 1 to be encoded.

One limitation of this paper is that we examined only two datasets related to a single disease. In future research, we plan to investigate the performance of LP on additional LOAD GWAS datasets as well as GWAS datasets from other diseases.

## Conclusions

Biomarker discovery in GWAS data is a challenging problem with the potential for many false positives and the lack of reproducibility across datasets. LP had excellent comparative performance among the four feature ranking methods we applied in this paper, based on the results of classification accuracy, reproducibility, biological validity, and running time. The LP method is effective in all of these performance measures across a range of experimental conditions, while the other methods tested are weak in at least one of these areas. These results provide support for including LP in the methods that are used to rank SNPs in high-dimensional GWAS datasets.

## Abbreviations

ADRC: Alzheimer Disease Research Center; AUC: Area under the (receiver operating characteristic) curve; kNN: k-nearest-neighbor classifier; GWAS: Genome-wide association study; LOAD: Late-onset Alzheimer’s disease; LP: Label propagation; MAF: Minor allele frequency; SLR: Sparse logistic regression; SNP: Single nucleotide polymorphism; SWRF: Sigmoid weighted ReliefF; TGen: Translational Genomics Research Institute.

## Competing interests

The authors declare that they have no competing interests.

## Authors’ contributions

MES designed the study, performed the experiments and drafted the manuscript, MIK and SV designed the study and edited the manuscript, MMB edited the manuscript. All authors read and approved the final manuscript.

## Supplementary Material

Additional file 1 **Top 25 SNPs as ranked by each algorithm (chi squared, SWRF, LR, and LP) on two LOAD datasets (TGen and ADRC).** Each SNP rsID is listed with the associated chromosome and gene, as well as any connection to LOAD in the literature. Click here for file
