# Supplementary material for: The application of network label propagation to rank biomarkers in genome-wide Alzheimer’s data
Source: BMC Genomics. 2014 Apr 14;15:282. doi: 10.1186/1471-2164-15-282 (PMC4234455; doi:10.1186/1471-2164-15-282)
Supplement: Additional file 1 — Top 25 SNPs as ranked by each algorithm (chi squared, SWRF, LR, and LP) on two LOAD datasets (TGen and ADRC). Each SNP rsID is listed with the associated chromosome and gene, as well as any connection to LOAD in the literature. [file 1471-2164-15-282-S1.docx]

**Table A1.** Top 25 SNPs as ranked by LP (α=0.25) on genome-wide TGen data. 14 show association with LOAD.

| **Rank** | **rsID** | **Gene** | **Chr** | **Comment** |
| --- | --- | --- | --- | --- |
| 1 | rs7412 | APOE | 19 | APOE risk allele determined by rs7412 and rs429358 [1] |
| 2 | rs4420638 | APOC | 19 | In strong linkage disequilibrium with APOE SNPs [2] |
| 3 | rs429358 | APOE | 19 | APOE risk allele determined by rs7412 and rs429358 [3] |
| 4 | rs10824310 | PRKG1 | 10 | Significant association with LOAD [4] |
| 5 | rs12162084 | - | 16 | Significant association with LOAD [5] |
| 6 | rs17330779 | NRCAM | 7 | Associated with axonal degeneration in LOAD [6] |
| 7 | rs7077757 | RBM20 | 10 | Meta-analysis of multiple studies showed association [7] |
| 8 | rs10115381 | - | 9 | - |
| 9 | rs4356530 | - | 17 | Association found in another analysis of TGen data  [8] |
| 10 | rs6717497 | - | 2 | - |
| 11 | rs2913719 | - | 5 | Association in systematic meta-analysis of AD [9] |
| 12 | rs12476792 | - | 2 | - |
| 13 | rs17169622 | BMPER | 7 | - |
| 14 | rs1038891 | LRRC4C | 11 | SNP associated with LOAD in genome-wide analysis [10] |
| 15 | rs10499687 | VWC2 | 7 | - |
| 16 | rs7335085 | - | 13 | - |
| 17 | rs16974268 | SLCO3A1 | 15 | - |
| 18 | rs10996618 | - | 10 | SNP selected in logistic regression analysis [11] |
| 19 | rs950922 | ALPL | 1 | - |
| 20 | rs17151710 | - | 5 | Found in meta-analysis of 3 studies [12] |
| 21 | rs9934599 | IL34 | 16 | - |
| 22 | rs473367 | - | 9 | SNP may interact with APOE to affect LOAD [13] |
| 23 | rs4862146 | - | 4 | Glycoprotein buildup affects nerve cells in the brain [14] |
| 24 | rs6013406 | ZFP64 | 20 | - |
| 25 | rs1712417 | TMEM87A | 15 | - |

**Table A2.** Top 25 SNPs as ranked by LP (α=0.25) on genome-wide ADRC data. 10 show association with LOAD.

| **Rank** | **rsID** | **Gene** | **Chr** | **Comment** |
| --- | --- | --- | --- | --- |
| 1 | rs439401 | APOE | 19 | In strong LD with rs7412 and rs429358 [15] |
| 2 | rs5157 | APOC4 | 19 | In strong LD with other APOC risk SNPs [16] |
| 3 | rs2075650 | TOMM40 | 19 | Predictive of longevity of LOAD patients [17, 18] |
| 4 | rs445925 | - | 19 | Showed LOAD association in African-American cohort [19] |
| 5 | rs157182 | ZNF433 | 19 | Other zinc finger proteins linked to LOAD [20] |
| 6 | rs283129 | PIN1 | 5 | PIN1 linked to neural apoptosis in LOAD [21, 22] |
| 7 | rs17428956 | - | 1 | - |
| 8 | rs11076978 | - | 16 | - |
| 9 | rs5749272 | NDRG1 | 22 | NDRG family linked to neuron development, LOAD [23, 24] |
| 10 | rs6754487 | - | 2 | - |
| 11 | rs439401 | - | 19 | Near APOE, associated with LOAD [25] |
| 12 | rs3738269 | IGFN1 | 1 | - |
| 13 | rs10106829 | LOC157273 | 8 | - |
| 14 | rs12520115 | - | 5 | - |
| 15 | rs17018886 | - | 2 | - |
| 16 | rs17821171 | - | 15 | - |
| 17 | rs523079 | - | 3 | - |
| 18 | rs2314221 | - | 2 | - |
| 19 | rs13059988 | - | 3 | - |
| 20 | rs356611 | - | 5 | - |
| 21 | rs10976056 | KDM4C | 9 | - |
| 22 | rs10489926 | PRG5 | 1 | Brain-specific protein linked to axonal health [26] |
| 23 | rs10489924 | PRG5 | 1 | Brain-specific protein linked to axonal health [26] |
| 24 | rs2712599 | - | 12 | - |
| 25 | rs10459209 | - | 12 | - |

**Table A3.** Top 25 SNPs as ranked by SLR on genome-wide TGen data. 7 show association with LOAD.

| **Rank** | **rsID** | **Gene** | **Chr** | **Comment** |
| --- | --- | --- | --- | --- |
| 1 | rs7412 | APOE | 19 | APOE risk allele determined by rs7412 and rs429358 [1] |
| 2 | rs429358 | APOE | 19 | APOE risk allele determined by rs7412 and rs429358 [3] |
| 3 | rs7662187 | PDGFC | 4 | - |
| 4 | rs10778921 | TMTC2 | 12 | - |
| 5 | rs7335085 | - | 13 | - |
| 6 | rs12162084 | - | 16 | Association found in another analysis of TGen data [5] |
| 7 | rs16923249 | - | 9 | - |
| 8 | rs6508182 | DCC | 18 | Implicated in axonal development [27] |
| 9 | rs4902299 | - | 14 | - |
| 10 | rs10510990 | - | 3 | - |
| 11 | rs16916338 | GABBR2 | 9 | Gene involved in neurotransmitters [28] |
| 12 | rs10894424 | NTM | 11 | Gene implicated in LOAD [29] |
| 13 | rs1728390 | - | 16 | - |
| 14 | rs4351927 | GPC5 | 13 | Involved in neuronal development [30] |
| 15 | rs16907781 | ZBTB10 | 8 | - |
| 16 | rs10871528 | - | 18 | - |
| 17 | rs7848622 | - | 9 | - |
| 18 | rs11846241 | EML5 | 14 | - |
| 19 | rs17044664 | - | 3 | - |
| 20 | rs6540253 | - | 16 | - |
| 21 | rs10176594 | - | 2 | - |
| 22 | rs7243005 | - | 18 | - |
| 23 | rs10740667 | - | 10 | - |
| 24 | rs10966006 | - | 9 | - |
| 25 | rs6824979 | MMRN1 | 4 | - |

**Table A4.** Top 25 SNPs as ranked by SLR on genome-wide ADRC data. 5 show association with LOAD.

| **Rank** | **rsID** | **Gene** | **Chr** | **Comment** |
| --- | --- | --- | --- | --- |
| 1 | rs429358 | APOE | 19 | APOE risk allele determined by rs7412 and rs429358 [3] |
| 2 | rs4420638 | APOC | 19 | In strong linkage disequilibrium with APOE SNPs [2] |
| 3 | rs7412 | APOE | 19 | APOE risk allele determined by rs7412 and rs429358 [1] |
| 4 | rs8083752 | LOC643542 | 18 | - |
| 5 | rs1978326 | MAGI2 | 7 | Gene associated with hoppocampal volume reduction in AD [25] |
| 6 | rs6015314 | APCDD1L-AS1 | 20 | - |
| 7 | rs17767748 | BTRC | 10 | - |
| 8 | rs11695991 | NEU2 | 2 | - |
| 9 | rs7210298 | - | 17 | - |
| 10 | rs12190755 | ZNF318 | 6 | Gene expression level linked to AD [31] |
| 11 | rs7606208 | SLC9A2 | 2 | - |
| 12 | rs9932776 | - | 16 | - |
| 13 | rs11680648 | DIRC3 | 2 | - |
| 14 | rs12100042 | - | 13 | - |
| 15 | rs12257119 | MYO3A | 10 | - |
| 16 | rs4147209 | - | 1 | - |
| 17 | rs17099379 | SYT16 | 14 | - |
| 18 | rs7009155 | - | - | - |
| 19 | rs801289 | - | 2 | - |
| 20 | rs10862184 | MYF5 | 12 | - |
| 21 | rs16846388 | SPATA16 | 3 | - |
| 22 | rs9299784 | KIAA1217 | 10 | - |
| 23 | rs1759320 | - | 10 | - |
| 24 | rs10507341 | - | 13 | - |
| 25 | rs2276754 | CCDC174 | 3 | - |

**Table A5.** Top 25 SNPs as ranked by chi squared on genome-wide TGen data. 6 show association with LOAD.

| **Rank** | **rsID** | **Gene** | **Chr** | **Comment** |
| --- | --- | --- | --- | --- |
| 1 | rs4420638 | APOC | 19 | In strong linkage disequilibrium with APOE SNPs [2] |
| 2 | rs7412 | APOE | 19 | APOE risk allele determined by rs7412 and rs429358 [3] |
| 3 | rs934745 | MAPK4 | 18 | - |
| 4 | rs429358 | APOE | 19 | APOE risk allele determined by rs7412 and rs429358 [3] |
| 5 | rs7079348 | C10orf11 | 10 | - |
| 6 | rs188429 | RCL1 | 9 | - |
| 7 | rs10824310 | PRKG1 | 10 | Significant association with LOAD [4] |
| 8 | rs6717497 | - | 2 | - |
| 9 | rs16938663 | STAU2 | 8 | - |
| 10 | rs3732443 | GXYLT2 | 3 | - |
| 11 | rs6453333 | - | 5 | - |
| 12 | rs12041702 | - | 1 | - |
| 13 | rs17048190 | - | 2 | - |
| 14 | rs2968848 | - | 7 | - |
| 15 | rs16909497 | - | 10 | - |
| 16 | rs10499687 | VWC2 | 7 | - |
| 17 | rs17169622 | BMPER | 7 | - |
| 18 | rs41479848 | MBIP | 14 | Involved in LOAD-associated MAPK pathway [32] |
| 19 | rs3007246 | - | 13 | - |
| 20 | rs6429224 | RGS7 | 1 | Gene involved in brain signaling [33] |
| 21 | rs10845804 | - | 12 | - |
| 22 | rs12109727 | - | 5 | - |
| 23 | rs12476792 | - | 2 | - |
| 24 | rs6455005 | - | 6 | - |
| 25 | rs11804140 | FBXO28 | 1 | - |

**Table A6.** Top 25 SNPs as ranked by chi squared on genome-wide ADRC data. 10 show association with LOAD.

| **Rank** | **rsID** | **Gene** | **Chr** | **Comment** |
| --- | --- | --- | --- | --- |
| 1 | rs429358 | APOE | 19 | APOE risk allele determined by rs7412 and rs429358 [3] |
| 2 | rs4420638 | APOC | 19 | In strong linkage disequilibrium with APOE SNPs [2] |
| 3 | rs157582 | TOMM40 | 19 | Showed LOAD association in African-American cohort [19] |
| 4 | rs2075650 | APOE4 | 19 | Predictive of longevity of LOAD patients [17, 18] |
| 5 | rs7412 | APOE | 19 | APOE risk allele determined by rs7412 and rs429358 [3] |
| 6 | rs405509 | APOE | 19 | APOE promoter varies LOAD risk [34] |
| 7 | rs8106922 | TOMM40 | 19 | Meta-analysis finds significant association with LOAD [9] |
| 8 | rs26845 | ECI1 | 16 | - |
| 9 | rs12507679 | STAP1 | 4 | - |
| 10 | rs13132585 | STAP1 | 4 | - |
| 11 | rs157580 | TOMM40 | 19 | Associated with LOAD in Chinese population [35] |
| 12 | rs4496012 | - | 13 | - |
| 13 | rs8082842 | RAB31 | 18 | Gene involved in potential treatment [36] |
| 14 | rs9487940 | - | 6 | - |
| 15 | rs34276 | ACACB | 12 | - |
| 16 | rs4865859 | - | 5 | - |
| 17 | rs7985095 | - | 13 | - |
| 18 | rs16976268 | - | 18 | - |
| 19 | rs4796922 | - | 18 | - |
| 20 | rs16841336 | PYHIN1 | 1 | - |
| 21 | rs832156 | IGFN1 | 1 | - |
| 22 | rs9438881 | - | 1 | - |
| 23 | rs283129 | PIN1 | 5 | PIN1 linked to neural apoptosis in LOAD [21, 22] |
| 24 | rs4480661 |  | 13 | - |
| 25 | rs11985315 | TRAPPC9 | 8 | - |

**Table A7.** Top 25 SNPs as ranked by SWRF on genome-wide TGen data. 5 show association with LOAD.

| **Rank** | **rsID** | **Gene** | **Chr** | **Comment** |
| --- | --- | --- | --- | --- |
| 1 | rs7412 | APOE | 19 | APOE risk allele determined by rs7412 and rs429358 [3] |
| 2 | rs250857 | FSTL4 | 5 | - |
| 3 | rs9328529 | - | 9 | - |
| 4 | rs934745 | MAPK4 | 18 | - |
| 5 | rs11077058 | RBFOX1 | 16 | RBFOX1 linked to brain volume in older adults [37] |
| 6 | s17124810 | CBFA2T2 | 20 | - |
| 7 | rs1251059 | - | 12 | - |
| 8 | rs9908065 | - | 17 | - |
| 9 | rs13213247 | - | 6 | Significant association in meta-analysis of LOAD [38] |
| 10 | rs8112622 | - | 19 | - |
| 11 | rs2779556 | GABBR2 | 9 | Gene involved in neurological pathways [39] |
| 12 | rs188429 | RCL1 | 9 | - |
| 13 | rs8108780 | - | 19 | - |
| 14 | rs2796460 | TLE1 | 9 | - |
| 15 | rs16910463 | - | 9 | - |
| 16 | rs16915130 | GRM5 | 11 | Gene is a coreceptor for LOAD-related protein [40] |
| 17 | rs16967491 | - | 15 | - |
| 18 | rs200556 | - | 9 | - |
| 19 | rs250855 | FSTL4 | 5 | - |
| 20 | rs4394475 | - | 9 | - |
| 21 | rs10454604 | - | 13 | - |
| 22 | rs8006542 | FOXN3 | 14 | - |
| 23 | rs865505 | - | 12 | - |
| 24 | rs2712271 | - | 1 | - |
| 25 | rs17141368 | - | 7 | - |

**Table A8.** Top 25 SNPs as ranked by SWRF on genome-wide ADRC data. 2 show association with LOAD.

| **Rank** | **rsID** | **Gene** | **Chr** | **Comment** |
| --- | --- | --- | --- | --- |
| 1 | rs439401 | - | 19 | Significant LOAD association [15] |
| 2 | rs445925 | - | 19 | Located between APOE and APOC genes [41] |
| 3 | rs6434513 | - | 2 | - |
| 4 | rs17245472 | - | 16 | - |
| 5 | rs182662 | RAB23 | 6 | - |
| 6 | rs4494677 | - | 2 | - |
| 7 | rs16906827 | - | 10 | - |
| 8 | rs11108379 | LTA4H | 12 | - |
| 9 | rs12683673 | KDM4C | 9 | - |
| 10 | rs2712599 | - | 12 | - |
| 11 | rs9297095 | - | 6 | - |
| 12 | rs4270681 | - | 5 | - |
| 13 | rs12592188 | - | 15 | - |
| 14 | rs2442968 | - | 18 | - |
| 15 | rs2442966 | - | 18 | - |
| 16 | rs1834804 | - | 14 | - |
| 17 | rs16963657 | - | 13 | - |
| 18 | rs11820815 | - | 11 | - |
| 19 | rs1892786 | - | 11 | - |
| 20 | rs7004779 | KCNK9 | 8 | - |
| 21 | rs6669982 | NFIA | 1 | - |
| 22 | rs9493552 | - | 6 | - |
| 23 | rs11004700 | - | 10 | - |
| 24 | rs6678065 | NFIA | 1 | - |
| 25 | rs10095543 | - | 8 | - |

**Additional References**

1. Izaks Gj Fau - Gansevoort RT, Gansevoort Rt Fau - van der Knaap AM, van der Knaap Am Fau - Navis G, Navis G Fau - Dullaart RPF, Dullaart Rp Fau - Slaets JPJ, Slaets JP: **The association of APOE genotype with cognitive function in persons aged 35 years or older.**

2. Bertram L, Lange C, Mullin K, Parkinson M, Hsiao M, Hogan MF, Schjeide BM, Hooli B, Divito J, Ionita I, et al: **Genome-wide association analysis reveals putative Alzheimer's disease susceptibility loci in addition to APOE.** *Am J Hum Genet* 2008, **83:**623-632.

3. Izaks GJ, Gansevoort RT, van der Knaap AM, Navis G, Dullaart RP, Slaets JP: **The association of APOE genotype with cognitive function in persons aged 35 years or older.** *PLoS ONE* 2011, **6:**e27415.

4. Fallin M, Szymanski M, Wang R, Gherman A, Bassett S, Avramopoulos D: **Fine mapping of the chromosome 10q11-q21 linkage region in Alzheimer's disease cases and controls.** *neurogenetics* 2010, **11:**335-348.

5. Jiang X, Barmada MM, Cooper GF, Becich MJ: **A Bayesian Method for Evaluating and Discovering Disease Loci Associations.** *PLoS ONE* 2011, **6:**e22075.

6. Hu WT, Chen-Plotkin A, Arnold SE, Grossman M, Clark CM, Shaw LM, Pickering E, Kuhn M, Chen Y, McCluskey L, et al: **Novel CSF biomarkers for Alzheimer's disease and mild cognitive impairment.** *Acta Neuropathologica* 2010, **119:**669-678.

7. Shi H, Medway C, Bullock J, Brown K, Kalsheker N, Morgan K: **Analysis of Genome-Wide Association Study (GWAS) data looking for replicating signals in Alzheimer's disease (AD).** *Int J Mol Epidemiol Genet* 2010, **1:**53-66.

8. Jiang X, Barmada MM, Becich MJ: **Evaluating De Novo Locus-Disease Discoveries in GWAS Using the Signal-to-Noise Ratio.** *AMIA Annu Symp Proc* 2011, **2011:**617-624.

9. **The AlzGene Database** [<http://www.alzgene.org>]

10. Liu W, Ding J, Gibbs JR, Wang SJ, Hardy J, Singleton A: **A simple and efficient algorithm for genome-wide homozygosity analysis in disease.** *Mol Syst Biol* 2009, **5:**304.

11. Briones N, Dinu V: **Data mining of high density genomic variant data for prediction of Alzheimer's disease risk.** *BMC Med Genet* 2012, **13:**7.

12. Shi H: *Complementary Approaches to Analyse Genetic Data in Late Onset Alzheimer's Disease (LOAD).* University of Nottingham; 2012.

13. Jiang X, Neapolitan RE, Barmada MM, Visweswaran S, Cooper GF: **A fast algorithm for learning epistatic genomic relationships.** *AMIA Annu Symp Proc* 2010, **2010:**341-345.

14. Saarela J, von Schantz C, Peltonen L, Jalanko A: **A novel aspartylglucosaminuria mutation affects translocation of aspartylglucosaminidase.** *Hum Mutat* 2004, **24:**350-351.

15. Abraham R, Moskvina V, Sims R, Hollingworth P, Morgan A, Georgieva L, Dowzell K, Cichon S, Hillmer AM, O'Donovan MC, et al: **A genome-wide association study for late-onset Alzheimer's disease using DNA pooling.** *BMC Med Genomics* 2008, **1:**44.

16. Cervantes S, Samaranch L, Vidal-Taboada JM, Lamet I, Bullido MJ, Frank-García A, Coria F, Lleó A, Clarimón J, Lorenzo E, et al: **Genetic variation in APOE cluster region and Alzheimer's disease risk.** *Neurobiology of Aging* 2011, **32:**2107.e2107-2107.e2117.

17. Shi H, Belbin O, Medway C, Brown K, Kalsheker N, Carrasquillo M, Proitsi P, Powell J, Lovestone S, Goate A, et al: **Genetic variants influencing human aging from late-onset Alzheimer's disease (LOAD) genome-wide association studies (GWAS).** *Neurobiol Aging* 2012, **33:**1849 e1845-1818.

18. Deelen J, Beekman M, Uh HW, Helmer Q, Kuningas M, Christiansen L, Kremer D, van der Breggen R, Suchiman HE, Lakenberg N, et al: **Genome-wide association study identifies a single major locus contributing to survival into old age; the APOE locus revisited.** *Aging Cell* 2011, **10:**686-698.

19. Logue Mw SMVBN, et al.: **A comprehensive genetic association study of alzheimer disease in african americans.** *Archives of Neurology* 2011, **68:**1569-1579.

20. Li G, Jiang H, Chang M, Xie H, Hu L: **HDAC6 α-tubulin deacetylase: A potential therapeutic target in neurodegenerative diseases.** *Journal of the neurological sciences* 2011, **304:**1-8.

21. Butterfield DA, Abdul HM, Opii W, Newman SF, Joshi G, Ansari MA, Sultana R: **Pin1 in Alzheimer's disease.** *J Neurochem* 2006, **98:**1697-1706.

22. Driver JA, Lu KP: **Pin1: a new genetic link between Alzheimer's disease, cancer and aging.** *Curr Aging Sci* 2010, **3:**158-165.

23. Okuda T, Higashi Y, Kokame K, Tanaka C, Kondoh H, Miyata T: **Ndrg1-deficient mice exhibit a progressive demyelinating disorder of peripheral nerves.** *Mol Cell Biol* 2004, **24:**3949-3956.

24. Kalaydjieva L, Gresham D, Gooding R, Heather L, Baas F, de Jonge R, Blechschmidt K, Angelicheva D, Chandler D, Worsley P, et al: **N-myc downstream-regulated gene 1 is mutated in hereditary motor and sensory neuropathy-Lom.** *Am J Hum Genet* 2000, **67:**47-58.

25. Potkin SG, Guffanti G, Lakatos A, Turner JA, Kruggel F, Fallon JH, Saykin AJ, Orro A, Lupoli S, Salvi E, et al: **Hippocampal Atrophy as a Quantitative Trait in a Genome-Wide Association Study Identifying Novel Susceptibility Genes for Alzheimer's Disease.** *PLoS ONE* 2009, **4:**e6501.

26. Broggini T, Nitsch R, Savaskan NE: **Plasticity-related gene 5 (PRG5) induces filopodia and neurite growth and impedes lysophosphatidic acid- and nogo-A-mediated axonal retraction.** *Mol Biol Cell* 2010, **21:**521-537.

27. Harter PN, Bunz B, Dietz K, Hoffmann K, Meyermann R, Mittelbronn M: **Spatio-temporal deleted in colorectal cancer (DCC) and netrin-1 expression in human foetal brain development.** *Neuropathol Appl Neurobiol* 2010, **36:**623-635.

28. Sherry ST, Ward MH, Kholodov M, Baker J, Phan L, Smigielski EM, Sirotkin K: **dbSNP: the NCBI database of genetic variation.** *Nucleic Acids Res* 2001, **29:**308-311.

29. Pan Y, Wang KS, Aragam N: **NTM and NR3C2 polymorphisms influencing intelligence: family-based association studies.** *Prog Neuropsychopharmacol Biol Psychiatry* 2011, **35:**154-160.

30. Baranzini SE, Wang J, Gibson RA, Galwey N, Naegelin Y, Barkhof F, Radue EW, Lindberg RL, Uitdehaag BM, Johnson MR, et al: **Genome-wide association analysis of susceptibility and clinical phenotype in multiple sclerosis.** *Hum Mol Genet* 2009, **18:**767-778.

31. Bossers K, Wirz KT, Meerhoff GF, Essing AH, van Dongen JW, Houba P, Kruse CG, Verhaagen J, Swaab DF: **Concerted changes in transcripts in the prefrontal cortex precede neuropathology in Alzheimer's disease.** *Brain* 2010, **133:**3699-3723.

32. Munoz L, Ammit AJ: **Targeting p38 MAPK pathway for the treatment of Alzheimer's disease.** *Neuropharmacology* 2010, **58:**561-568.

33. Zhang JH, Barr VA, Mo Y, Rojkova AM, Liu S, Simonds WF: **Nuclear localization of G protein beta 5 and regulator of G protein signaling 7 in neurons and brain.** *J Biol Chem* 2001, **276:**10284-10289.

34. Bizzarro A, Seripa, D., Acciarri, A. Matera, M.G., Pilotto, A., Tiziano, F.D., Brache, C., et al.: **The complex interaction between APOE promoter and AD: an Italian case study.** *European Journal of Human Genetics* 2009, **17:**7.

35. Ma XY, Yu JT, Wang W, Wang HF, Liu QY, Zhang W, Tan L: **Association of TOMM40 polymorphisms with late-onset Alzheimer's disease in a Northern Han Chinese population.** *Neuromolecular Med* 2013, **15:**279-287.

36. Polhner JH, DE): **Diagnostic and therapeutic use of a rab family gtp-binding protein for neurodegenerative diseases.** In *Book Diagnostic and therapeutic use of a rab family gtp-binding protein for neurodegenerative diseases* (Editor ed.^eds.). City: Evotec Neurosciences GmbH (Schnackenburgallee 114, Hamburg, DE); 2006.

37. Kohannim O, Hibar DP, Stein JL, Jahanshad N, Hua X, Rajagopalan P, Toga AW, Jack CR, Jr., Weiner MW, de Zubicaray GI, et al: **Discovery and Replication of Gene Influences on Brain Structure Using LASSO Regression.** *Front Neurosci* 2012, **6:**115.

38. Shi H, Medway C, Brown K, Kalsheker N, Morgan K: **Using Fisher's method with PLINK 'LD clumped' output to compare SNP effects across Genome-wide Association Study (GWAS) datasets.** *Int J Mol Epidemiol Genet* 2011, **2:**30-35.

39. Williams C, Mehrian Shai R, Wu Y, Hsu YH, Sitzer T, Spann B, McCleary C, Mo Y, Miller CA: **Transcriptome analysis of synaptoneurosomes identifies neuroplasticity genes overexpressed in incipient Alzheimer's disease.** *PLoS ONE* 2009, **4:**e4936.

40. Um JW, Kaufman AC, Kostylev M, Heiss JK, Stagi M, Takahashi H, Kerrisk ME, Vortmeyer A, Wisniewski T, Koleske AJ, et al: **Metabotropic glutamate receptor 5 is a coreceptor for Alzheimer abeta oligomer bound to cellular prion protein.** *Neuron* 2013, **79:**887-902.

41. Jun G, Vardarajan BN, Buros J, Yu C, Hawk MV, Dombroski BA, Crane PK, Larson EB, Mayeux R, Haines JL, et al: **Comprehensive Search for Alzheimer Disease Susceptibility Loci in the APOE Region.** *Arch Neurol* 2012**:**1-10.
